# Design of a homelessness-focused suicide prevention program

**DOI:** 10.1192/j.eurpsy.2024.1624

**Published:** 2024-08-27

**Authors:** F. Calvo, C. Giralt, R. Alfranca, X. Solench-Arco, I. Forcada, S. Font-Mayolas

**Affiliations:** ^1^Departament de Pedagogia, Institut de Recerca sobre Qualitat de Vida; ^2^ Institut Català de la Salut, Centre d’Atenció Primària Blanes 2; ^3^ Institut Català de la Salut, Centre d’Atenció Primària Santa Clara; ^4^ Universitat de Girona; ^5^Departament de Psicologia, Institut de Recerca sobre Qualitat de Vida, Girona, Spain

## Abstract

**Introduction:**

This project proposes a program for the promotion of mental health and prevention of suicidal behavior among individuals experiencing homelessness, with the aim of reducing suicidal ideation and suicide mortality within this vulnerable population.

**Objectives:**

The project aims to implement an evidence-based program to reduce suicidal ideation and suicide mortality among homeless individuals. This will be achieved through two phases: a review of scientific literature and the development of the program in collaboration with experts and homeless individuals.

**Methods:**

The first phase of the project involved a review of scientific literature to identify the most effective content and programs for improving mental health and preventing suicide. These findings were adapted for application in the program and for dissemination to professionals who will directly engage with individuals experiencing homelessness.

In the second phase, the program content was designed in collaboration with experts and validated through the input of educational professionals. Additionally, individuals experiencing homelessness actively participated in the creation of materials and the definition of the approach to be utilized. Subsequently, a four-hour training was provided to professionals working in specialized homeless shelters to guide groups of individuals experiencing homelessness.

**Results:**

The program consists of the following components:

- Training and Awareness: Workshops and campaigns to reduce the stigma surrounding suicide.
- Early Detection and Risk Assessment: Staff training in recognizing suicide indicators and risk assessment protocols.
- Psychological and Social Interventions: Crisis teams, individual and group therapy.
- Access to Services: Mobile mental health clinics and collaborations with healthcare professionals.
- Ongoing Support and Monitoring: Temporary housing programs and support groups.

**Conclusions:**

The proposed program seeks to mitigate the risk of suicide among individuals experiencing homelessness through a comprehensive approach. The collaboration of experts and homeless individuals ensures that the solutions are appropriate and effective. The implementation of this program has the potential to make a significant difference in promoting mental health and preventing suicide within this vulnerable population.

**Disclosure of Interest:**

None Declared

